# Slip-Ups in the Diagnosis of Cardiac Amyloidosis: A Case Fatality in Point

**DOI:** 10.7759/cureus.22458

**Published:** 2022-02-21

**Authors:** Marissa Hobocan, Ayesha Shaik, Amjad Saad, Oisharya Dasgupta, Abhishek Jaiswal

**Affiliations:** 1 Internal Medicine, University of Connecticut School of Medicine, Farmington, USA; 2 Cardiology, Hartford Hospital, Hartford, USA; 3 Pathology, Hartford Hospital, Hartford, USA; 4 Advanced Heart Failure, Heart Transplant and Pulmonary Hypertension, Hartford Hospital, Hartford, USA

**Keywords:** urine electrophoresis., serum protein, cardiac mri, breast amyloidosis, cardiac amyloidosis

## Abstract

This case report illustrates a tragic example of a "missed diagnosis" of amyloid light-chain (AL) amyloidosis with cardiac involvement that led to progressive heart failure and the ultimate death of the patient. It had a rather atypical presentation in terms of cardiac imaging, although there were certain highly suspicious clinical features, cardiac and otherwise. It also illustrates the importance of selecting the most appropriate assays to establish (or rule out) the presence of monoclonal immunoglobulin consistent with AL amyloidosis, which has a poor clinical prognosis, as unfortunately demonstrated in this case.

## Introduction

Cardiac amyloidosis is increasingly recognized as an important and underdiagnosed cause of heart failure, conduction abnormalities, and arrhythmias. Although it has become easier to diagnose cardiac amyloidosis with growing experience, the diagnostic workup can still be confusing and sometimes results in delayed diagnosis or misdiagnosis, which, in turn, might delay treatment and lead to poor outcomes. In this report, we describe a case where slip-ups in diagnostic evaluation resulted in a delayed diagnosis and fatality from cardiac amyloid light-chain (AL) amyloidosis. We also outline potential strategies to prevent such outcomes.

## Case presentation

**Figure 1:**
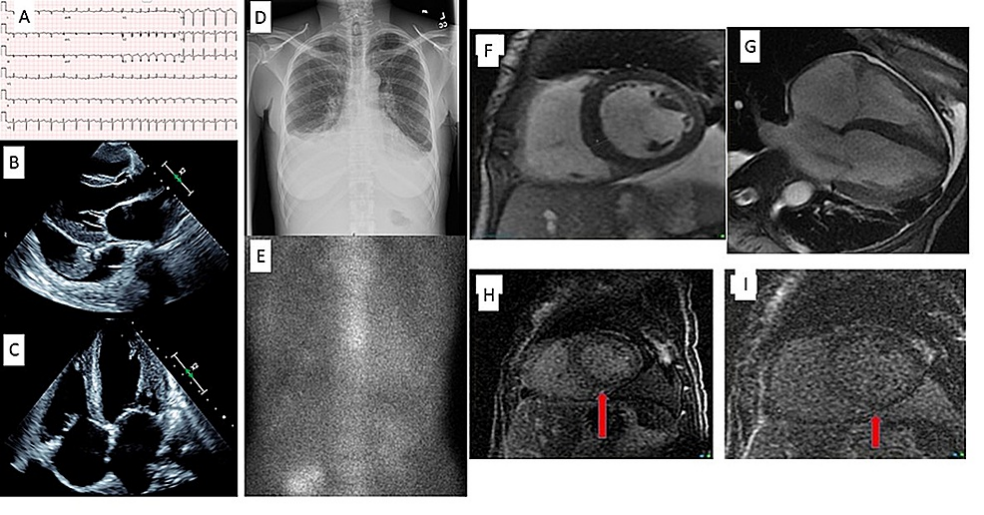
Cardiac imaging studies (A) EKG showed sinus tachycardia with a run of SVT with normal voltage in the precordial leads. (D) Chest X-ray showed normal heart size with pulmonary vascular congestion with small bilateral pleural effusions. (B, C) The echocardiogram revealed normal left ventricular wall thickness and indexed mass with bi-atrial dilation. (E) PYP imaging showed a lack of myocardial uptake. (F-I) CMR showed normal left ventricular wall thickness in the basal short-axis cine view without LGE; however, the retrospective analysis revealed difficulties nulling (arrows) the myocardium EKG: electrocardiogram; SVT: supraventricular tachycardia; PYP: pyrophosphate; CMR: cardiac magnetic resonance imaging; LGE: late gadolinium enhancement

A 62-year-old female with known iron deficiency anemia and symptomatic bilateral carpel tunnel syndrome diagnosed in 2013 and conservatively managed presented with progressive dyspnea with associated fatigue, orthopnea, and peripheral edema for over three months. An EKG on presentation showed sinus tachycardia with a run of supraventricular tachycardia (SVT) and an old anterolateral infarct with normal voltage in precordial leads, while chest X-ray demonstrated normal heart size with increased pulmonary vascular congestion and small pleural effusions (Figure 1). Cardiac biomarkers were notable for an elevated N-terminal pro-B-type natriuretic peptide (NT-proBNP, 2,959 pg/mL) and normal troponin I (<0.30 ng/mL). During hospitalization, an echocardiogram revealed severe left ventricular systolic dysfunction with ejection fraction (EF) <20% with normal left ventricular wall thickness (0.8 cm) and indexed mass and normal end-diastolic diameter (5.0 cm), moderate right ventricular systolic dysfunction, and bi-atrial dilation (Figure 1). No prior echocardiogram was available for comparison. Due to the presence of moderate-severe mitral regurgitation secondary to apical tethering of subvalvular apparatus of the posterior leaflet, the diastolic function could not be interpreted and no strain pattern was reported. Left and right heart catheterization (LHC/RHC) showed normal coronary arteries without obstruction and a cardiac index (CI) of 2.2 L/min/kg^2^. Cardiac MRI (CMR) and a 99m Technetium-pyrophosphate (PYP) scan were subsequently obtained as part of the initial evaluation for newly detected non-ischemic cardiomyopathy. CMR showed a normal left ventricular end-diastolic wall thickness of 1.3 cm without late gadolinium enhancement (LGE) and was read as a normal study, while PYP scan showed no increased myocardial tracer uptake by quantitative scoring (Figure 1).

Serum protein electrophoresis with immunofixation (SPIE) was unremarkable. However, urine protein electrophoresis was abnormal (62 mg/dL) with a monoclonal peak suggestive of a monoclonal gammopathy. Urine immunofixation was ordered, but never obtained or followed up. Serum-free light chain (SFLC) assay was also never pursued as part of the initial evaluation. Moreover, since the patient did not meet the CRAB (increased calcium level, renal dysfunction, anemia, and destructive bone lesions) criteria for multiple myeloma, a bone marrow biopsy was deferred as it was felt that AL amyloidosis would be an unlikely cause of such profound heart failure in the absence of an abnormal CMR and a normal SPIE. A diagnosis of monoclonal gammopathy of undetermined significance (MGUS) was entertained instead. Of note, this conclusion was made by the treating medicine and cardiology teams without input from hematology/oncology. Due to episodes of atrial and ventricular tachycardia, an implantable cardioverter-defibrillator (ICD) was recommended but refused by the patient. She was discharged on introductory doses of goal-directed medical therapies.

The patient's condition continued to decline with worsening fatigue, generalized weakness, weight loss, and dyspnea with activities of daily living. Despite these progressive symptoms, along with intolerance to medical management secondary to lower range blood pressure and the need for recurrent outpatient infusion clinic visits for aggressive diuresis, she refused hospitalization due to the ongoing coronavirus disease 2019 (COVID-19) pandemic. Approximately eight months after her first hospitalization, she required admission for the management of clinical cardiogenic shock and fluid overload (RHC: CI: 1.6 L/min/kg^2^). She required escalating doses of inopressors (norepinephrine, epinephrine, dopamine) and an intravenous diuretic regimen but her condition failed to improve.

**Figure 2:**
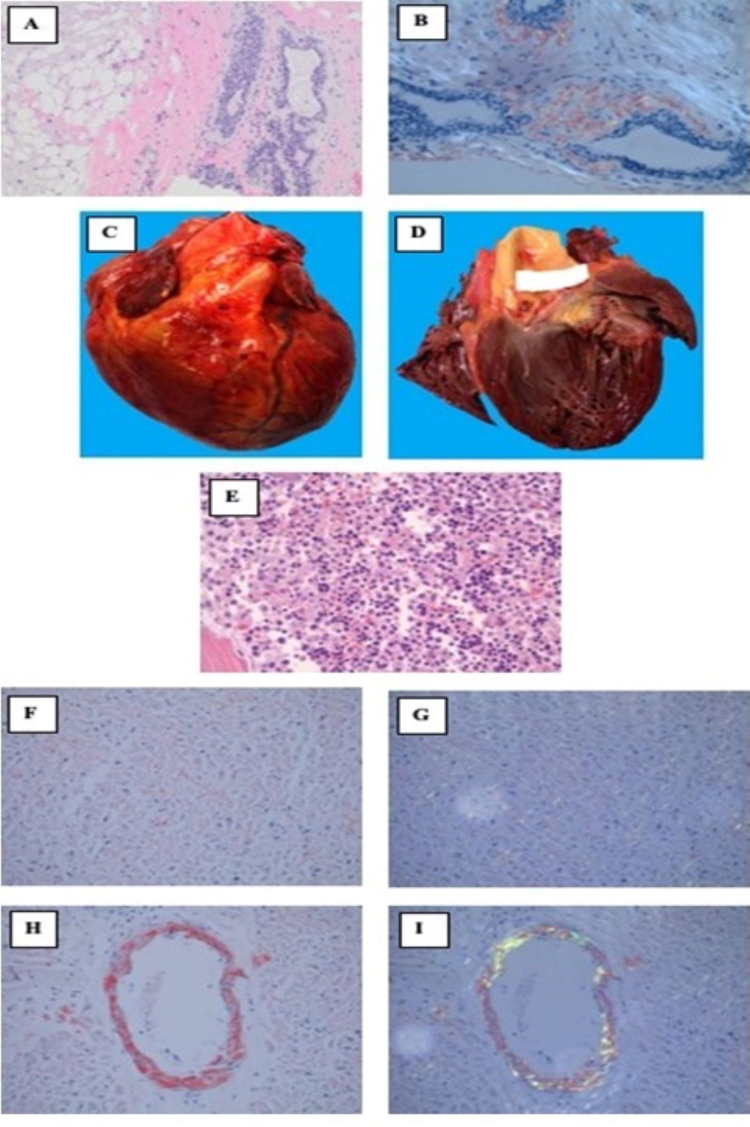
Breast biopsy and cardiac and bone marrow autopsy (A) Congo red-stained section of the breast demonstrates extensive stromal amyloid deposits of pink amorphous material. (B) Apple-green birefringence of amyloid material in the breast under polarized light. (C, D) Gross photographs of the heart demonstrate cardiomegaly and thickened myocardium. (C) The microscopic section of bone marrow reveals hypercellular marrow and abnormal plasma cell infiltrate. (F, G) Congo red-stained sections of myocardium showing both peri-myocytic and vascular deposits of pink amorphous material. (G, H) Apple-green birefringence of amyloid material in the myocardium under polarized light

Considering her clinical decline, an urgent workup for advanced heart failure therapies was initiated. Given a prior abnormal mammogram and a family history of breast cancer, she underwent a breast biopsy, which showed extensive stromal pink amorphous material on Congo red staining, confirming amyloid deposits (Figure 2). These findings prompted an oncology consultation and a reconsideration of the diagnosis of AL amyloidosis (Figure 2). A repeat echocardiogram again demonstrated severe left ventricular systolic dysfunction; however, it now also showed mild concentric hypertrophy (interventricular septal thickness of 1.1 cm versus 0.8 cm previously) and moderate dilation with global hypokinesis. SFLC assay showed elevated kappa and lambda levels (22.1 and 880 mg/L with a ratio of 0.03). Urine immunofixation revealed abnormal free lambda light chains. Bone marrow and cardiac biopsies could not be performed due to a rapid clinical decline and subsequent death. The autopsy showed hypercellular bone marrow with abnormal plasma cell infiltrate, cardiomegaly with thickened myocardium (cardiac weight of 415 grams; right ventricular wall thickness was 0.5 cm, left ventricular wall thickness was 2.0 cm, and interventricular septum thickness was 2.3 cm as compared to 1.1 cm as found during the most recent echocardiography a month prior), and Congo red stain showing widespread myocardial peri-myocytic and vascular deposits of pink amorphous material with apple-green birefringence under polarized light demonstrating amyloid. Widespread deposits of amyloid in the lungs were found as well (Figure 2).

## Discussion

Over the course of the last decade, cardiac amyloidosis has undergone a transition from a rare, underdiagnosed terminal disease with no established treatment to a more common, treatable condition; hence, in this study, we aimed to highlight the importance of heightened clinical suspicion, prompt diagnosis, and treatment of this condition. AL amyloidosis results from the overproduction and misfolding of monoclonal immunoglobulin light chains and is one of the most common systemic amyloidosis with a rapidly progressive clinical course. Cardiac involvement is common and happens to be the cause of death in approximately half of the patients with AL amyloidosis. A delay in diagnosis or missed diagnosis of AL cardiac amyloidosis can be catastrophic as untreated cardiac involvement has only a median survival of <6 months. However, improvement in light-chain suppressive therapy has led to a marked improvement in outcomes over the past decade and timely institution of the light-chain suppression therapies can improve median survival to >4 years [1,2]. In comparison, wild-type transthyretin (ATTR) cardiac amyloidosis typically has a more insidious onset.

The gold standard diagnostic method of cardiac amyloidosis involves the identification of abnormal proteins deposited in the heart via endomyocardial biopsy, but this is invasive and can be problematic. Thus, in clinical practice, the diagnosis is typically made by non-invasive tests in suspected patients with clinical and supporting features during routine cardiac imaging. The presence of traditional imaging features on echocardiography and CMR can be supportive but are not specific enough to diagnose cardiac amyloidosis, or distinguish AL from ATTR. However, the incorporation of routine use of global longitudinal strain assessment during echocardiography might be helpful in improving its utility in the diagnosis of amyloidosis. A reduced global longitudinal left ventricular strain with relative apical sparing points to cardiac amyloidosis. In our case, we did not have myocardial strain imaging available. A subsequent software upgrade allowed us to re-evaluate echocardiographic images of our patient, which showed abnormally reduced longitudinal strain (around -2) without apical sparing.

The initial screening for AL amyloidosis involves a combination of serum and urine protein electrophoresis with immunofixation (SPIE/UPIE) and SFLC assays. If these test results are normal, then the next step would be dedicated cardiac imaging to evaluate for ATTR amyloidosis. Bone scintigraphy with technetium-labeled bisphosphonates has high sensitivity and specificity for ATTR in the presence of normal serum and urine protein studies [1,2].

The common CMR features that suggest a diagnosis of cardiac amyloidosis are as follows: (1) increased wall thickness, (2) a non-coronary, often subendocardial, pattern of LGE, both within the atrium and ventricular myocardium. Cardiac amyloidosis with normal wall thickness and without LGE has been described with only mild disease. Our case is unique in that the wall thickness was within normal range and LGE was largely absent despite clinically advanced features. It is possible that our patient might have had pre-existing dilated cardiomyopathy with secondary AL amyloidosis more recently. However, this remains unlikely due to the recent rapidly progressive heart failure progression and lack thereof in prior follow-ups, but an asymptomatic progressive dilated cardiomyopathy cannot be ruled out entirely in the absence of prior imaging. It appears likely that our patient may have had increased left ventricular thickness compared to her healthy state, but that was still within the normal range. In rare cases, patients may have severe heart failure with only a minimal increase in wall thickness; this may be due to extensive replacement of the myocardium by amyloid, with a reduction in cellular volume [3]. Even so, the presence of LGE usually represents advanced cardiac amyloidosis, which was, intriguingly, absent in our case.

It is possible that the lack of abnormal LGE could have been falsely absent due to the lack of contrast between minimal normal myocardium and amyloid in cases with extensive amyloidosis and minimal cellular volume. Such patients have abnormal T1 mapping with an increase in native T1 and increased extracellular volume fraction (ECV) representative of amyloid burden [2]. Unfortunately, we were unable to obtain T1 mapping due to the lack of facility. Recent technological developments have enabled T1 mapping that can be generated using the single breath-hold technique [4]. Importantly, the use of gadolinium contrast agent could be relatively contraindicated in cases of severe renal failure, which is common in patients with systemic AL amyloidosis, and therefore, T1 mapping is a useful tool for the diagnosis of cardiac amyloidosis. Moreover, measurement of native myocardial T1 and ECV values facilitates the risk stratification of patients with cardiac amyloidosis as native T1 values >1044 ms and ECV values >45% could be associated with mortality [5]. However, T2 values do not exhibit significant changes with this condition and, therefore, would not provide much diagnostic assistance [6].

AL amyloidosis resulting in heart failure without myocardial infiltration is not seen. Additionally, amyloid infiltration of the heart is required before light-chain cardiotoxicity can clinically manifest. Hence, it is unlikely that our patient had a completely normal CMR with clinical evidence of cardiac amyloidosis. Accordingly, we reexamined CMR imaging and noted a difficulty nulling the myocardium, which was initially discounted and attributed to motion artifact in the absence of increased wall thickness and no obvious LGE (Figure 1). In retrospect, CMR may have demonstrated an abnormal nulling pattern, but in the absence of any other features suggestive of amyloidosis, the initial interpretation of motion artifact seemed reasonable. It would be very unusual to see abnormal nulling in an otherwise normal MRI as nulling is a late finding, of low sensitivity, and rarely if ever seen in patients without LGE [7].

On the other hand, failure to null the myocardium is highly specific for infiltrative pathologies such as amyloidosis. Moreover, symptomatic carpal tunnel syndrome in the absence of traditional risk factors and newly progressive non-ischemic cardiomyopathy should have been considered as highly indicative of infiltrative cardiomyopathy before discounting the difficulty in nulling the myocardium as a motion artifact. Therefore, this case stresses the need for proper attention to nulling the myocardium and extracellular volume assessment even when wall thickness and LGE are unrevealing, as well as the need for clinical correlation in patients with suspected amyloidosis. The reversal of nulling, which is said to be characteristic of amyloidosis, happens due to a combination of (a) myocardial amyloid deposition causing an increase in extracellular space resulting in gadolinium accumulation and shortening of the myocardial T1 time and (b) systemic amyloid load extracting the gadolinium from the blood pool, thereby causing relative prolongation of the blood T1 time. This additive two-fold effect essentially causes myocardial T1 to become shorter than blood T1 following gadolinium administration. Thus, an early reversed nulling pattern could possibly predict higher myocardial amyloid load and higher systemic amyloid load [5,7].

A normal SPIE that measures intact (whole) immunoglobulins, only a light chain (in high concentrations), or rarely, only a heavy chain does not rule out light-chain producing pathology. However, one-sixth of the patients may have a free light chain only, in which case serum protein electrophoresis (SPEP) is negative. A more sensitive SFLC assay can measure even small amounts of free kappa and lambda light chains in the blood and calculate a kappa/lambda ratio to help detect, monitor, and prognosticate conditions associated with the increased production of free light chains. The combination of SPIE, UPIE, and SFLC testing has a combined sensitivity of 99% for the identification of AL amyloidosis [8,9]. Nevertheless, in 1-2% of cases, routine laboratory tests are unable to detect the presence of free light chains either due to the relatively low burden or the sensitivity of the assay itself [8]. Altogether, to increase sensitivity and avoid false-negative cases, repeat serum and urine studies should be obtained in two to four weeks in such events. Furthermore, if the light chain’s conformation is such that it is prone to misfold, AL amyloid deposits can occur, regardless of whether the light chain is produced in large quantities or if the plasma cell clone itself is particularly aggressive. Accordingly, despite such atypical findings, our patient with high clinical suspicion should have undergone further exploration before her abnormal studies were attributed to MGUS. If clinical suspicion remains high, then tissue biopsy should be pursued. Additionally, this patient should have had a longitudinal hematology/oncology follow-up. Alternatively, in the absence of any clinical evidence of AL or myeloma, and with a normal SFLC ratio, it is highly unlikely that hematology would have performed a bone marrow biopsy anyway.

Beyond these slip-ups, postponement of the planned breast biopsy for abnormal mammogram findings due to the COVID-19 pandemic also caused a delay in cardiac AL amyloidosis diagnosis. Eventual breast biopsy during repeat hospitalization for heart transplantation workup showed amyloidosis, which prompted re-evaluation and a diagnosis of AL cardiac amyloidosis in this case. It is noteworthy that breast amyloidosis is predominantly the result of the AL variant and is often found as a late presentation of systemic disease and mandates a thorough evaluation [10].

**Figure 3:**
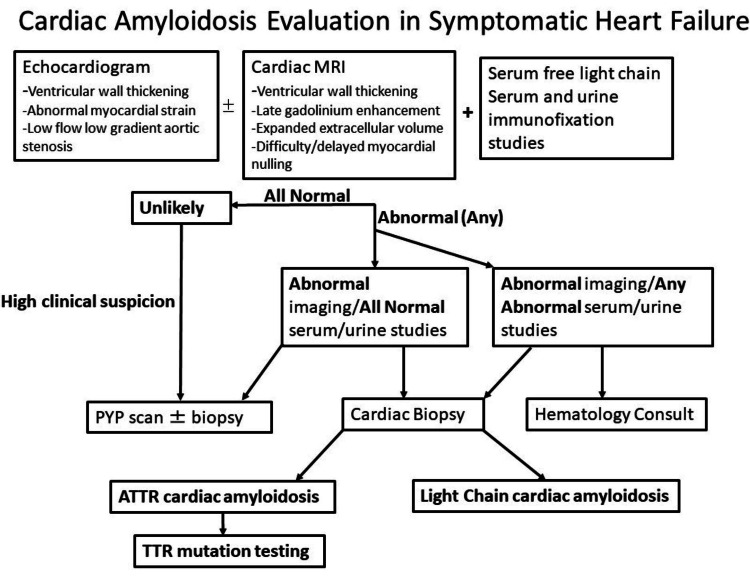
Approach to cardiac amyloidosis evaluation in symptomatic heart failure MRI: magnetic resonance imaging; PYP: pyrophosphate; ATTR: transthyretin

Currently, myocardial strain imaging, cardiac MRI, and biopsy are available only in a few centers. Given the recent interest in providing care for suspected cardiac amyloidosis cases locally, many centers are developing cardiac amyloidosis management programs. Against this backdrop, our case highlights the importance of developing multimodality imaging locally or collaborating with a center with such capabilities to minimize delayed or misdiagnosis of cardiac amyloidosis. We present a brief outline of an approach that we propose for suspected cardiac amyloidosis evaluation in patients with symptomatic heart failure (Figure 3).

## Conclusions

Our case highlights several potential gaps that one might encounter during the evaluation of cardiac amyloidosis, including the approach to the evaluation of monoclonal expansion with an incomplete set of investigations and misinterpretation of imaging. Moreover, this case stresses the importance of a thorough multidisciplinary evaluation of any abnormal protein study and the need for tissue biopsy in cases where clinical suspicion remains high. Unless a concerted effort is made to look into cardiac amyloidosis, it will remain an overlooked and under-recognized condition, to the detriment of these patients.
